# Inhibition of Heme Peroxidases by Melamine

**DOI:** 10.1155/2012/416062

**Published:** 2012-07-18

**Authors:** Pattaraporn Vanachayangkul, William H. Tolleson

**Affiliations:** Division of Biochemical Toxicology, National Center for Toxicological Research, US Food and Drug Administration, 3900 NCTR Road, Jefferson, AR 72079, USA

## Abstract

In 2008 melamine-contaminated infant formula and dairy products in China led to over 50,000 hospitalizations of children due to renal injuries. In North America during 2007 and in Asia during 2004, melamine-contaminated pet food products resulted in numerous pet deaths due to renal failure. Animal studies have confirmed the potent renal toxicity of melamine combined with cyanuric acid. We showed previously that the solubility of melamine cyanurate is low at physiologic pH and ionic strength, provoking us to speculate how toxic levels of these compounds could be transported through the circulation without crystallizing until passing into the renal filtrate. We hypothesized that melamine might be sequestered by heme proteins, which could interfere with heme enzyme activity. Four heme peroxidase enzymes were selected for study: horseradish peroxidase (HRP), lactoperoxidase (LPO), and cyclooxygenase-1 and -2 (COX-1 and -2). Melamine exhibited noncompetitive inhibition of HRP (*K*_*i*_  9.5 ± 0.7 mM), and LPO showed a mixed model of inhibition (*K*_*i*_  14.5 ± 4.7 mM). The inhibition of HRP and LPO was confirmed using a chemiluminescent peroxidase assay. Melamine also exhibited COX-1 inhibition, but inhibition of COX-2 was not detected. Thus, our results demonstrate that melamine inhibits the activity of three heme peroxidases.

## 1. Introduction

In 2007, the incidence of nephrotoxic renal failure of cats and dogs caused the recall of 1,177 lots of pet food products in the USA that were contaminated with melamine, cyanuric acid, and related triazine compounds [1]. The US Food and Drug Administration (FDA) and the US Department of Agriculture identified triazine contaminants in wheat gluten, rice protein, and corn gluten raw materials imported from China that were used as ingredients in pet food products [2]. In 2008, over 50,000 children exposed to foods manufactured using melamine-contaminated milk powder were hospitalized with renal injuries, and at least 6 died [3–5].

**Figure 1:**
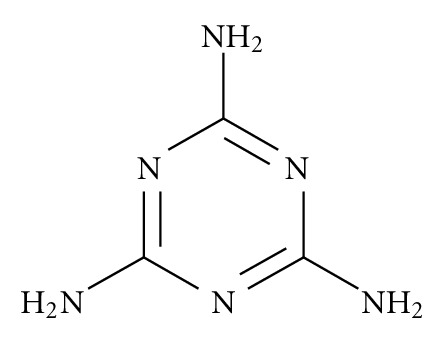
Structure of melamine (2,4,6-triamino-1,3,5-triazine [108-78-1)].

Melamine is a high-production industrial chemical used in the manufacture of thermosetting plastics, flame retardants, and fertilizers [6]. Melamine is an organic base with a 1,3,5-triazine skeleton (Figure 1) and a high nitrogen content (66% w/w). Ingredients used in food manufacturing that have higher total nitrogen levels achieve proportionally higher market prices because the total nitrogen level is used as an indirect index of the protein content. It has been alleged that melamine was added intentionally to raw materials sold by distributors to food manufacturers to elevate the apparent nitrogen content of those ingredients.

The toxicity of melamine has not been studied in humans; however, several studies have demonstrated renal crystal formation and kidney failure in rats, fish, cats, pigs, and monkeys following administration of melamine and cyanuric acid [7–13]. The solubility of melamine cyanurate is lower at physiological pH and ionic strength than it is in the acidic gastric compartment [14], provoking us to speculate how these two compounds could be absorbed and distributed throughout the body without crystallizing until passing into the renal filtrate. Wang et al. [15] developed a sensitive chemiluminescent method for detecting melamine in milk that measures the intensities of chemiluminescent emissions released in reactions between myoglobin and luminol. These authors showed that the chemiluminescent intensities of these reactions were diminished in proportion to the concentration of melamine. In support of their model, they also reported that the heme-dependent absorption maximum of myoglobin at 409 nm was decreased in the presence of melamine. Both of these observations are consistent with the formation of a myoglobin-melamine complex. We hypothesized that melamine might be partially sequestered by heme proteins *in vivo*, limiting the formation of insoluble complexes with cyanuric acid. We also speculated that melamine could interfere with the activity of some heme enzymes. In the current study, we tested this hypothesis by evaluating the effects of melamine on the catalytic activities of four heme peroxidase enzymes: horseradish peroxidase, lactoperoxidase, cylooxygenase-1, and cyclooxygenase-2.

## 2. Materials and Methods

### 2.1. Chemicals

Peroxidase type VI-A from horseradish (HRP), lactoperoxidase (LPO), sheep cyclooxygenase-1 (COX-1), human cyclooxygenase-2 (COX-2), 2,2′-azino-bis(3-ethylbenzothiazoline-6-sulfonic acid) diammonium salt (ABTS), potassium iodide (KI), sodium phosphate monobasic monohydrate (NaH_2_PO_4_), Bis-Tris (2,2-*bis*(hydroxymethyl)-2,2′,2′′-nitrilotriethanol), Trizma hydrochloride (*tris*(hydroxymethyl)aminomethane hydrochloride), 30% hydrogen peroxide (H_2_O_2_), bovine serum albumin (BSA), bovine hemin, *N*,*N*,*N*′,*N*′-tetramethyl-p-phenylenediamine (TMPD), Tween 20, cyanuric acid, and melamine were purchased from Sigma-Aldrich (St. Louis, MO, USA). Sodium arachidonate was purchased from Nu-Chek-Prep (Elysian, MN, USA). Phosphate buffered saline (PBS) was purchased from Fisher Scientific (Houston, TX, USA).

### 2.2. Instrument

A Synergy 4 Multi-Detection Microplate Reader with the dual-reagent dispense module from Biotek Instrument, Inc. (Winooski, VT, USA) was used for the enzyme activity assays described below.

### 2.3. Horseradish Peroxidase Assays

**Figure 2:**
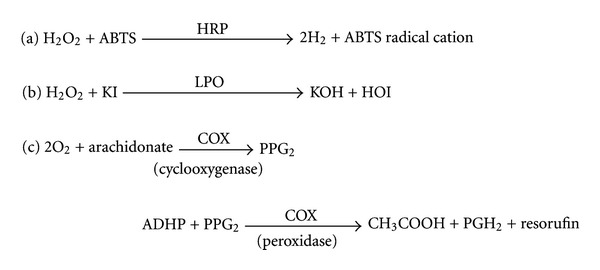
The enzymatic reactions of (a) horseradish peroxidase, (b) lactoperoxidase, and (c) cylooxygenases.

The experimental procedure was modified from a spectrophotometric assay for peroxidases (EC 1.11.1.7) [16, 17] that utilizes the reaction mechanism given in Figure 2(a). 50 mM of NaH_2_PO_4_, pH 5.0 was used to prepare 7 concentrations of ABTS (1.53, 3.05, 6.1, 9.2, 12.2, 15.25, and 18.4 mM). HRP was diluted with 0.025% BSA and 0.5% Tween 20 in 50 mM NaH_2_PO_4_, pH 6.0 to obtain 0.5 unit/mL HRP. 20 mM of melamine was dissolved in PBS as a stock solution and used to prepare 4 concentrations of melamine (5, 10, 15, and 20 mM). 30% H_2_O_2_ was diluted with water to obtain 0.3% H_2_O_2_.

205 *μ*L of ABTS solution, 25 *μ*L of melamine, and 10 *μ*L of 0.5 unit/mL of HRP were added to each well of 96-well UV plate, mixed, and the UV absorption at 405 nm was monitored until constant. The enzyme-catalyzed reaction was started by adding 10 *μ*L of 0.3% H_2_O_2_ and the change in absorbance at 405 nm (oxidized ABTS as a product, *ε* = 36.8 mM^−1 ^cm^−1^) was recorded for 60 sec. Blank reactions included 10 *μ*L enzyme dilution buffer instead of 0.5 unit/mL HRP.

Final concentrations for the 250 *μ*L reactions were 43 mM NaH_2_PO_4_, 0.012% H_2_O_2_, 1.25–15 mM ABTS, 0.1–2 mM melamine, and 0.02 unit/mL HRP. Negative control reactions contained cyanuric acid instead of melamine. All reactions were performed at room temperature.

### 2.4. Lactoperoxidase Assays

The spectrophotometric assay method (see Figure 2 for reaction) developed by Kussendrager and van Hooijdonk [18] was adapted for 96-well microplates. The concentration of LPO was determined by UV absorption at 412 nm (*ε* = 112.3 mM^−1 ^cm^−1^). 100 mM Bis-Tris pH 6.0 was used to prepare 7 concentrations of KI (5, 10, 25, 50, 75, 100, and 200 mM), 15 mM H_2_O_2_ and 20 mM melamine as a stock solution. Melamine stock solution was diluted to 3 concentrations (8, 12, and 20 mM). LPO was diluted to obtain 30 nM in 0.025% BSA in 100 mM Bis-Tris buffer pH 6.0.

150 *μ*L of melamine (replaced by 150 *μ*L of 100 mM Bis-Tris buffer pH 6.0 for blank reactions) and 20 *μ*L of KI were added to each well. 20 *μ*L of 30 nM LPO and 10 *μ*L of 15 mM H_2_O_2_, respectively, were dispensed using automated injectors, reactions were mixed for 1 sec, and the change in UV absorbance at 350 nm due to hypoiodite (HOI) formed as a product (*ε* = 60 M^−1 ^cm^−1^) was recorded for the initial 20 sec period at room temperature [19, 20]. Each 200 *μ*L reaction contained 100 mM Bis-Tris buffer pH 6.0, 0.75 mM H_2_O_2_, 30 nM LPO, 9–15 mM melamine, and 0.5–20 mM KI. Cyanuric acid replaced melamine in negative control reactions.

### 2.5. Chemiluminescent Assays for Horseradish Peroxidase and Lactoperoxidase

The chemiluminescent peroxidase substrate for ELISA kits was purchased from Sigma-Aldrich (product code CPS2-60). The procedure given in the technical bulletin was followed as given. All reactions were incubated at room temperature. Solutions containing 2 unit/mL lactoperoxidase and 8 unit/mL horseradish peroxidase were prepared in 0.075% BSA in 50 mM NaH_2_PO_4_, pH 6.0. Melamine was dissolved in PBS to provide a 20 mM stock solution used to prepare 9 dilutions of melamine in PBS (1.2–25 mM). 90 *μ*L of melamine and 10 *μ*L of enzyme were added to each well of 96-well microplate (white bottom plate) in triplicate. Then, 100 *μ*L of substrate was added to each well, the microplates were incubated in the dark for 5 min after 5 seconds shaking, and the steady-state chemiluminescent intensity was measured for each well. The total volume was 200 *μ*L per well, and final concentrations were 0.5625–11.25 mM melamine and 0.1 unit/mL LPO or 0.4 unit/mL HRP. Using this system, the intensities of the steady-state chemiluminescent emissions from these reactions are proportional to the rates of the enzyme-catalyzed reactions occurring in these samples.

### 2.6. Cyclooxygenase Assays

A fluorescence-based COX activity assay kit (Cayman Chemical Company, Ann Arbor, MI, USA) was used in this study following the procedure given by the manufacturer. In this method, the peroxidase activity of COX catalyzes the oxidation and *N*-deacetylation of ADHP to form resorufin, a highly fluorescent product.

A COX-1 working solution was prepared by diluting the enzyme to 400 unit/mL of COX-1 using the assay buffer provided with the kit (100 mM Tris-HCl, pH 8.0). Similarly, COX-2 was diluted to 20, 100, and 200 unit/mL with assay buffer. A heme cofactor working solution was prepared by diluting 40 *μ*L of the reagent provided in the assay kit with 960 *μ*L assay buffer. Immediately prior to conducting an experiment, the contents of an ADHP substrate vial provided with the kit were dissolved with 100 *μ*L DMSO then diluted with 900 *μ*L assay buffer. A 2.0 mM arachidonic acid substrate solution was also prepared fresh immediately before use according to the manufacturer's instructions using the supplied reagent, potassium hydroxide, and assay buffer. A 20 mM melamine stock solution was prepared in PBS and diluted to 0.5–20 mM.

Sample wells contained 150 *μ*L of assay buffer, 10 *μ*L of heme, 10 *μ*L of fluorometric substrate (ADPH), 10 *μ*L of COX, and 10 *μ*L of melamine. 10 *μ*L PBS was substituted for melamine in positive control wells. 20 *μ*L assay buffer replaced 10 *μ*L COX, and 10 *μ*L arachidonic acid in background wells. Reactions were initiated by adding 10 *μ*L of arachidonic acid substrate solution to each of the sample and positive control wells rapidly, incubating 60 seconds, then recording the fluorescent intensities for each well (EX 535/EM 590). The final concentrations in each sample well were 0.025–1 mM melamine, 1 unit/mL COX-1 or 1, 5, and 10 unit/mL COX-2, and 0.1 mM arachidonic acid. The fluorescence intensities from background wells were subtracted from those for sample and positive control wells to obtain corrected fluorescence values. Resorufin concentrations in sample and control wells were determined by comparison to standard curves that were prepared in each 96-well assay plate using 8 concentrations of resorufin (0, 0.1, 0.2, 0.4, 0.8, 1.2, 1.6, and 2.0 *μ*M) diluted with assay buffer. Reaction velocities were calculated by dividing resorufin concentrations by the incubation time (1 minute).

### 2.7. Data Analysis

All experiments were prepared in triplicate and enzyme kinetic parameters were obtained from GraphPad Prism version 5.01 software (GraphPad Software Inc. San Diego, CA, USA). Comparisons using two-tailed ANOVA were considered significant at *P* < 0.05. Dunnett's multiple comparisons test was applied to compare treated samples to controls.

## 3. Results and Discussion

**Figure 3:**
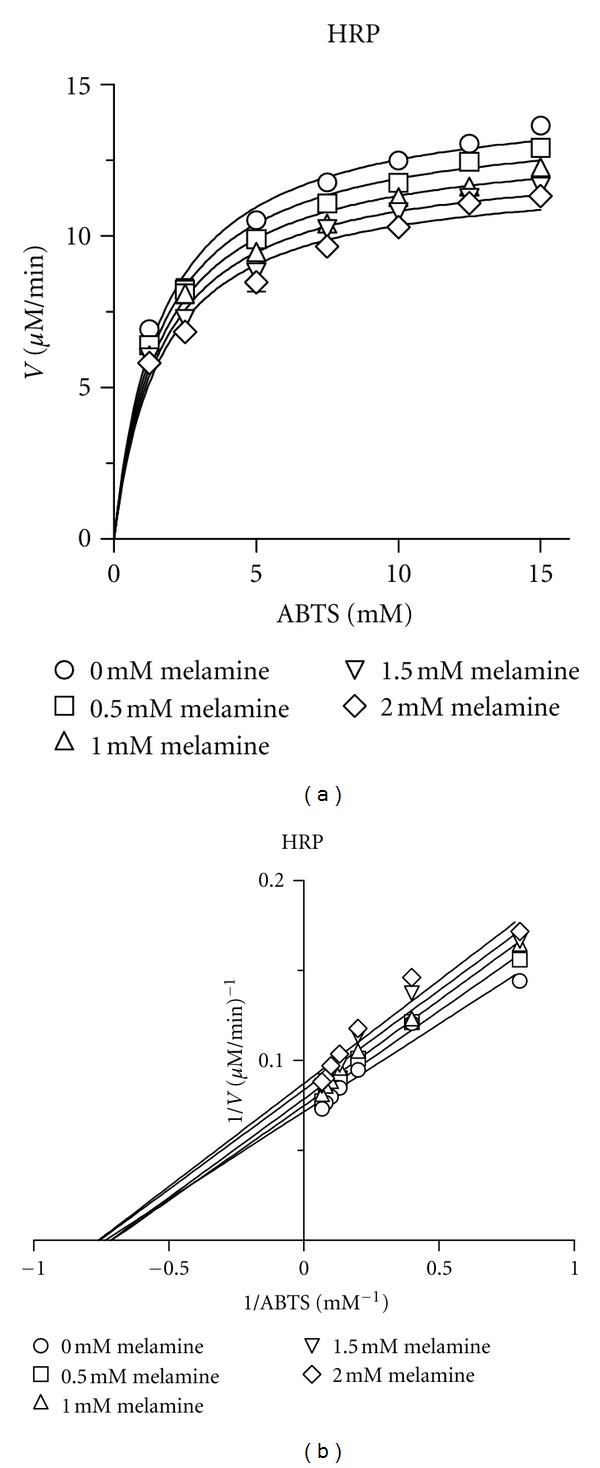
(a) Plots of velocity (*V*) of oxidized ABTS formation versus ABTS concentration (ABTS) with melamine concentration ranged from 0.5–2 mM. (b) Lineweaver-Burk plots of velocity (*V*) of oxidized ABTS formation versus ABTS concentration (ABTS) with melamine concentration ranged from 0.5–2 mM showing noncompetitive inhibition.

**Figure 4:**
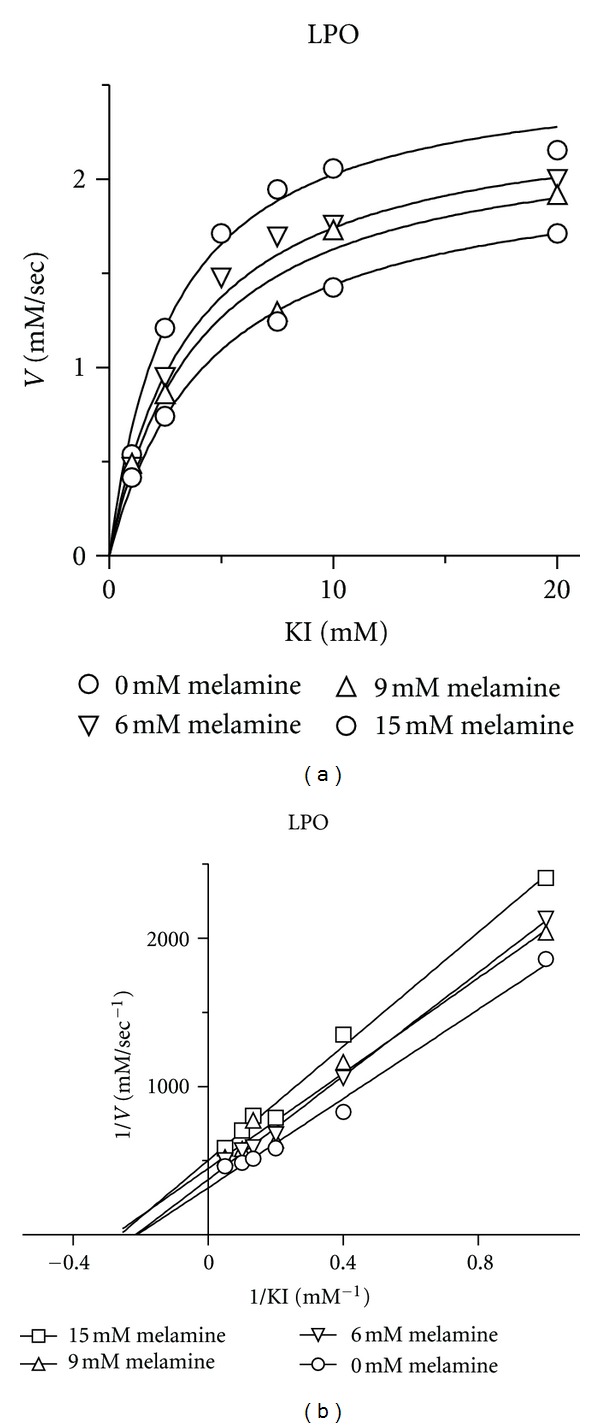
(a) Michaelis-Menton plots of the velocity of the lactoperoxidase-catalyzed reaction versus KI concentration with 6–15 mM melamine. (b) Lineweaver-Burk plots showing mixed model inhibition (primarily competitive) of lactoperoxidase by melamine.

**Figure 5:**
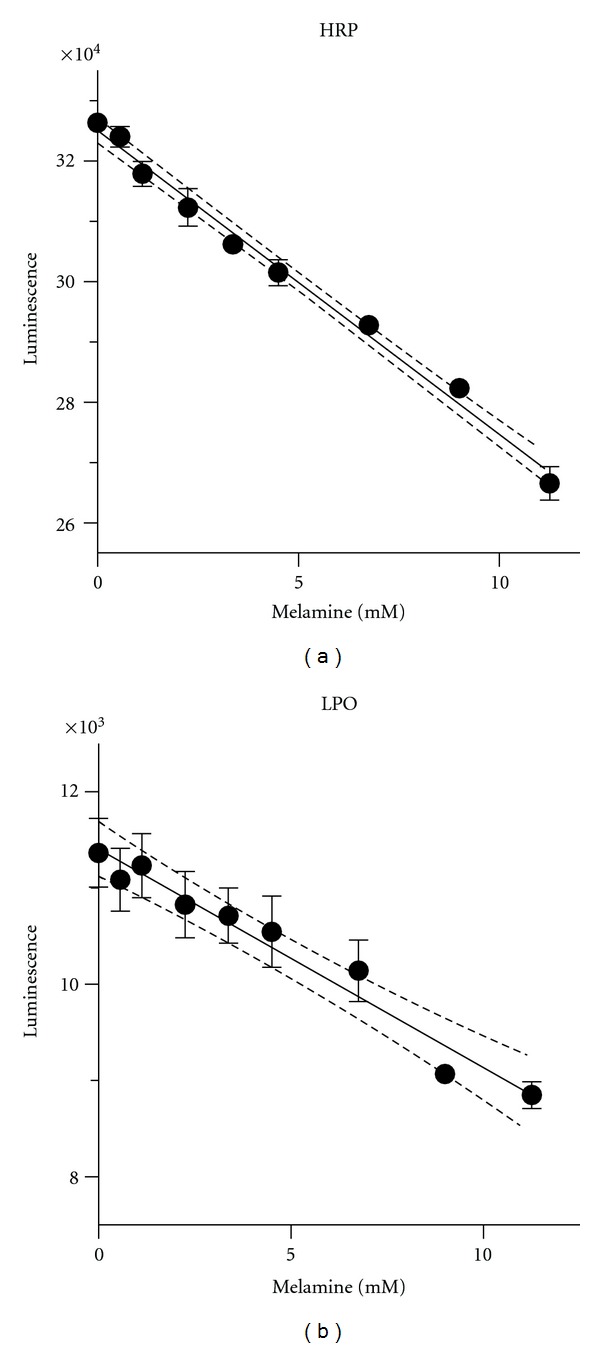
Plots of chemiluminescent intensity for reactions catalyzed by (a) horseradish peroxidase or (b) lactoperoxidase showing inhibition by melamine. Dotted lines depict 95% confidential bands for linear trends.

Melamine exhibited noncompetitive inhibition of horseradish peroxidase using the H_2_O_2_/ABTS assay, with *V*_max⁡_ = 14.63 ± 0.15 *μ*M/min, *K*_*m*_ = 1.67 ± 0.06 mM, and *K*_*i*_ = 9.5 ± 0.7 mM (Figure 3 and Table 1). A noncompetitive mechanism of inhibition implies that melamine binding does not compete with ABTS substrate binding but decreases the rate of catalytic turnover. No evidence of inhibition was observed in reactions in which cyanuric acid (1,3,5-triazine-2,4,6-triol) was substituted for melamine (data not shown). Melamine exhibited a mixed-model inhibition of lactoperoxidase (primarily competitive) using the H_2_O_2_/KI iodoperoxidase assay (Figure 4 and Table 1) with *V*_max⁡_ = 2.60 ± 0.10 mM/sec, *K*_*m*_ = 2.9 ± 0.4 mM, and *K*_*i*_ = 15 ± 5 mM. A mixed model of inhibition implies that melamine interferes with ABTS binding and also impairs the reaction velocity. Inhibition of horseradish peroxidase and lactoperoxidase by melamine was confirmed using the chemiluminescent peroxidase assay method (Figure 5). Linear trends between luminescent intensity and melamine concentration were evident for both enzymes. Cyanuric acid failed to inhibit horseradish peroxidase and lactoperoxidase using the chemiluminescent assay (data not shown). Unfortunately, enzyme kinetic constants and inhibition constants could not be calculated using this method because the supplier declined to provide the identity and concentration of the chemiluminescent substrate.

**Figure 6:**
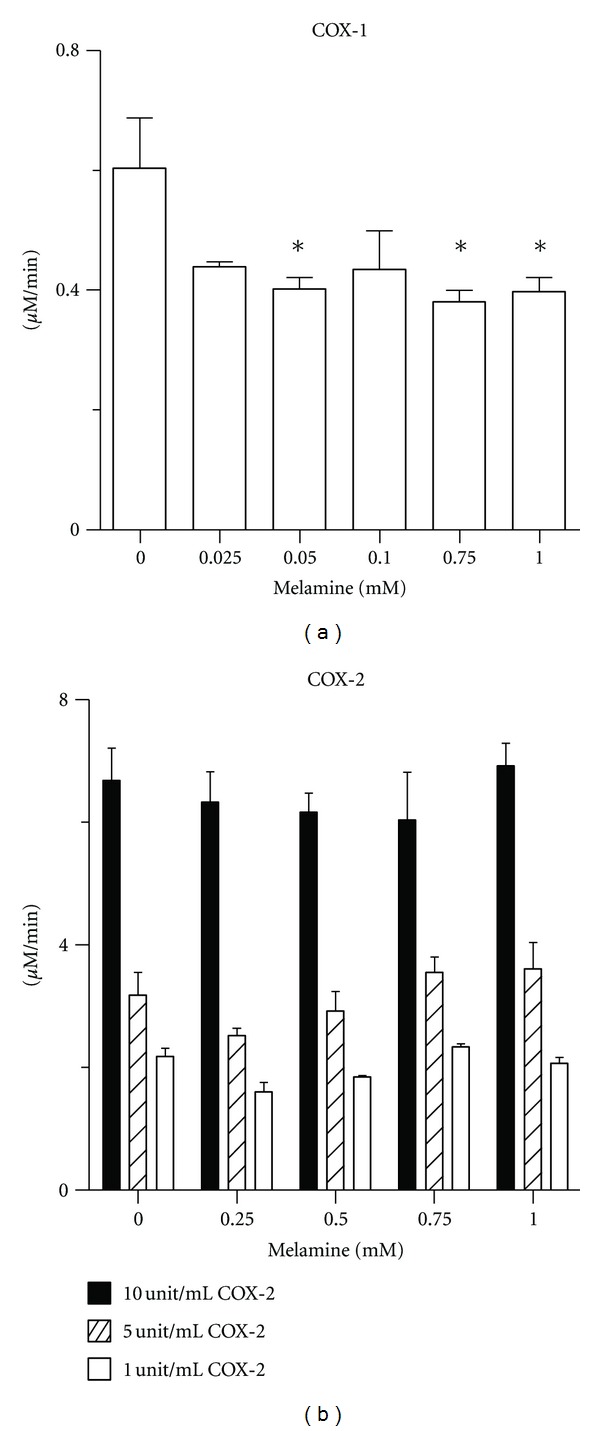
Effects of melamine on peroxidase activity of (a) COX-1 and (b) COX-2.

COX-1 and COX-2 are prostaglandin H synthases (EC 1.14.99.1) that convert arachidonic acid (AA) to prostaglandin H_2_ (PGH_2_) in two steps [21]. In the first step, the cyclooxygenase activity of COX acts as a dioxygenase to catalyze the incorporation of two moles of molecular oxygen to arachidonic acid (AA) to form prostaglandin G_2_ (PGG_2_), a reactive 15-hydroperoxy-9,10-endoperoxide. COX acts as a peroxidase in the second step in which a cosubstrate molecule serves as an electron donor to reduce the PGG_2_ hydroperoxyl group which then becomes the hydroxyl group of prostaglandin H_2_. We evaluated the effects of melamine on the peroxidase activity of COX-1 and COX-2 using 10-acetyl-3,7-dihydroxyphenoxazine (ADHP; Amplex Red) as the electron donor substrate. Melamine exhibited a significant concentration-dependent trend for COX-1 inhibition using the fluorescent assay method (Figure 6(a)). These data showed that COX-1 activity was inhibited in reactions containing 0.05–1.00 mM melamine. Inhibition of COX-2 was not apparent using this method.

Recent pharmacokinetic studies showed that melamine administered orally to Sprague-Dawley rats was absorbed almost completely (98.1% bioavailability) and then excreted rapidly (*t*_1/2_ 194 ± 38 min), primarily via filtration through the kidneys [9]. However, repeated exposure of lambs to high doses of melamine (2, 10, 30, or 100 mg/kg) or to 100 mg/kg melamine plus 100 mg/kg cyanuric acid for 60 days led to increasing melamine levels in the serum (167–267 *μ*g/kg max), liver (158–412 *μ*g/kg max), longissimus dorsi and gluteal muscles (227–374 *μ*g/kg max), and kidney (347–808 *μ*g/kg max) [22]. The tissue levels of melamine observed in animals do not reach levels required for lactoperoxidase or COX-1 inhibition under the conditions described in his report, although it could be speculated that somewhat higher melamine levels might occur within the microenvironment of renal tubule cells. Nonetheless, our results show that melamine interferes with the catalytic activity of three of the four heme enzymes tested, demonstrating intermolecular interactions between melamine and HRP, LPO, and COX-1. Studies by Wang [15] implicated interactions between melamine and another heme protein, myoglobin. Therefore, it will be important to determine whether other proteins present in plasma and/or urine may sequester melamine and/or cyanuric acid. Melamine- or cyanuric binding-proteins may inhibit crystal formation outside of the urinary tract and could influence the adsorption, transport, and retention of these compounds.

## Figures and Tables

**Table 1:** Enzyme kinetic parameters for horseradish peroxidase and lactoperoxidase.

	HRP^a^	LPO^b^
*V*_max⁡_	14.63 ± 0.15 *μ*M/min⁡	2.60 ± 0.10 mM/sec
*K*_*m*_	1.67 ± 0.06 mM	2.9 ± 0.4 mM
*K*_*i*_	9.5 ± 0.7 mM	15 ± 5 mM
Type of inhibition	Noncompetitive	Mixed model (primarily competitive)

^a^ *V*_max⁡_ and *K*_*m*_ for HRP refer to ABTS as the substrate. ^b^ *V*_max⁡_ and *K*_*m*_ for LPO refer to potassium iodide as the substrate.

## Abbreviations

ABTS: 2,2′-Azino-bis(3-ethylbenzthiazoline-6-sulfonic acid)
KI: Potassium iodide
HOI: Hypoiodite
AA: Arachidonic acid
PGG_2_: Prostaglandin G_2_
PGH_2_: Prostaglandin H_2_
ADHP: 10-Acetyl-3, 7-dihydroxyphenoxazine
TMPD: *N*,*N*,*N*′,*N*′-Tetramethyl-p-phenylenediamine.
